# Daytime circadian patterns of exhaled volatile organic compounds in adults without and with type 1 and type 2 diabetes: protocol for an exploratory observational study

**DOI:** 10.1136/bmjopen-2025-113892

**Published:** 2026-04-08

**Authors:** Cléo Nicolier, Marc Rothenbühler, Maria Luisa Balmer, Lilian Witthauer

**Affiliations:** 1 Department of Diabetes, Endocrinology, Nutritional Medicine and Metabolism, Inselspital, University of Bern, Bern University Hospital, Bern, Switzerland; 2 Diabetes Center Berne, Bern, Switzerland; 3 Institute for Infectious Diseases, University of Bern, Bern, Switzerland

**Keywords:** General diabetes, Diabetes Mellitus, Type 2, Microbiota, Clinical Protocols

## Abstract

**Introduction:**

Circadian regulation modulates metabolic and hormonal processes throughout the day, yet it remains unclear whether these diurnal fluctuations are reflected in exhaled volatile organic compound (VOC) profiles and whether such temporal patterns differ between individuals with and without diabetes. Previous breath analysis studies in diabetes have shown heterogeneous results, which may reflect differences in analytical approaches and the lack of standardised sampling times.

**Methods and analysis:**

This prospective, single-centre observational study examines daytime VOC dynamics from 08:00 to 16:00 among adults without diabetes, and individuals with type 1 diabetes or type 2 diabetes. 60 participants will complete one in-person visit with repeated breath measurements using a BreathSpec^®^ gas chromatography–ion mobility spectrometry system (GC-IMS) device, capillary glucose testing, body composition assessment, questionnaires, and oral and stool microbiota sampling. A standardised breakfast is provided; subsequent meals follow structured timing but are not standardised. The primary outcome is temporal variation in VOC intensities. Secondary outcomes include between-group differences and associations with glucose levels, body composition and microbiota composition. Analyses will use established GC–IMS tools and exploratory multivariate approaches.

**Ethics and dissemination:**

Ethics approval was granted by the Ethics Committee of the Canton of Bern (BASEC 2023-01143). Results will be shared via peer-reviewed publications, conferences and lay summaries.

**Trial registration number:**

NCT05984979.

**STRENGTHS AND LIMITATIONS OF THIS STUDY**

- Controlled yet pragmatic single-day design enables assessment of daytime variation in exhaled volatile organic compounds (VOCs) under near-real-world conditions.
- Inclusion of participants without diabetes, with type 1 diabetes and with type 2 diabetes enables cross-group comparison of metabolic influences.
- Multimodal sampling (breath VOCs, glucose, body composition, oral and stool microbiota) supports integrative analyses.
- Exploratory nature and modest sample size limit formal inference; findings will primarily inform future powered studies.
- Dependence on a single gas chromatography–ion mobility spectrometry system instrument introduces vulnerability to device downtime and scheduling delays.

## Introduction

 Breath volatile organic compounds (VOCs) arise from endogenous metabolism, microbial activity and environmental exposures, and have been evaluated as non-invasive markers in metabolic and inflammatory contexts.^1^,^4^ In diabetes research, altered levels of VOCs such as ethanol, isopropanol, dimethyl sulfide, isoprene and pentanal have been reported in individuals with type 1 diabetes (T1D) compared with individuals without diabetes.^5^ Other studies suggest associations between exhaled acetone, methyl nitrate, methanol, propane and circulating glucose,^6^ and hypoglycaemia-linked breath signatures have been observed.^7 8^ However, these findings are not uniform. Some investigations identified glucose–acetone correlations, whereas others did not.^9 10^ Heterogeneity in analytical platforms, diet, gut microbiota composition and function, metabolic status and sampling procedures likely contributes to these inconsistencies.^11^ Despite these limitations, breath VOC analysis holds potential as a non-invasive adjunct to existing biomarkers, offering real-time metabolic information relevant to glycaemic monitoring, early detection of dysglycaemic states, disease stratification and potentially early identification of metabolic dysregulation in populations at risk for or with undiagnosed type 2 diabetes (T2D),^11 12^ though superiority over established markers has not yet been demonstrated.

Circadian physiology may also influence breath VOC patterns. Hormonal rhythms, eating behaviour, nutritional composition and microbial metabolism vary across the day.^11^,^14^ Breath studies in respiratory medicine have shown time-of-day differences in specific exhaled metabolites.^15^ Yet, in diabetes research, sampling times are rarely standardised, and circadian effects are seldom considered.^5^,^79 10^ If temporal variation exists, ignoring it may obscure clinically meaningful signals; if minimal, sampling may be more flexible than assumed.

This study investigates whether breath VOC profiles show daytime variation under controlled but pragmatic conditions. We focus on an 8-hour window (08:00–16:00) with fasting at entry, a standardised breakfast and structured breath-sampling intervals. The aim is to characterise temporal patterns in exhaled VOCs in adults with T1D and T2D and those without diabetes, and to explore associations with glucose, body composition and oral and stool microbiota composition. Findings from this exploratory work may support more informed design of breath-based metabolic monitoring studies.

To address these objectives, we designed an observational protocol with predefined sampling frequency, dietary timing and analytic steps. The sections below describe the study procedures and statistical plan.

## Methods and analysis

### Study design and setting

This prospective, single-centre observational study is conducted in the clinical study facilities at the Diabetes Center Berne (DCB), Switzerland. Each participant completes one 8-hour study visit. The study aims to estimate daytime breath VOC dynamics rather than test a formal intervention or treatment effect.

Recruitment began in 2023, with data collection completed for three participants. The study has been paused since June 2024 due to device unavailability and resumed in December 2025. No interim analyses have been conducted to date. Enrolment is expected to conclude by October 2026, with primary data analysis completed within approximately 6 months thereafter.

### Participants and eligibility

Three adult groups are enrolled: individuals without diabetes, those with T1D and those with T2D. Participants must be ≥18 years old. Individuals with T1D must have a diagnosis for ≥1 year and use multiple daily injections or insulin pump therapy. The no-diabetes group requires no clinical diagnosis of diabetes and glycated haemoglobin (HbA1c) <6.5%. The T2D group requires a documented medical diagnosis with HbA1c>6.5% and treatment with oral antidiabetic medication and/or insulin therapy. Recruitment aims for approximate balance by sex and by body mass index (BMI) category (<25 kg/m² vs ≥25 kg/m²) as specified in the protocol, but groups are not randomised or strictly stratified by these variables.

Key exclusions (applied across all groups) include pregnancy or breastfeeding; smoking within 6 months; chronic pulmonary or intestinal disease (eg, asthma, Chronic obstructive pulmonary disease (COPD), Inflammatory bowel disease (IBD)); coeliac disease; lactose or fructose intolerance; acute infection; current use of inhaled medicines; antibiotic treatment within the previous 4 months; and daily alcohol consumption >4 units.

Potential confounders (eg, recent diet, sleep, physical activity, recent or mild intercurrent infections not meeting exclusion criteria, medication changes and environmental exposures) are documented via diaries and questionnaires for descriptive adjustment in analyses. Socioeconomic factors are not systematically controlled in this exploratory protocol and are noted as limitations. All eligibility criteria are summarised in table 1.

**Table 1:** Eligibility criteria

Group	Criteria
All participants	Age ≥18 years; able to provide written informed consent
No diabetes	No clinical diabetes diagnosis; HbA1c <6.5%
Type 1 diabetes (T1D)	Documented T1D; insulin therapy (MDI or CSII) ≥1 year
Type 2 diabetes (T2D)	Documented T2D; HbA1c >6.5%; oral agents and/or insulin therapy
Exclusion (all groups)	Pregnancy/breastfeeding; smoking within 6 months; chronic pulmonary or intestinal disease (eg, asthma, COPD, IBD); coeliac disease; lactose or fructose intolerance; acute infection; current inhaled medicines; antibiotics within 4 months; alcohol >4 units/day

COPD, Chronic obstructive pulmonary disease; CSII, continuous subcutaneous insulin infusion; HbA1c, glycated hemoglobin; HbA1c, glycated haemoglobin; IBD, Inflammatory bowel disease; MDI, multiple daily injection.

### Recruitment and consent

Recruitment occurs through flyers, local clinics, doctors’ offices and online postings. Interested individuals complete an initial self-screen. The study team confirms eligibility. Written informed consent is obtained before procedures. Participants receive 100 CHF compensation.

### Study procedures

Participants are instructed to fast from midnight before the study visit and to abstain from alcohol for 24 hours prior to the visit. On the morning of the visit, they are asked to avoid using toothpaste and mouthwash for at least 1 hour before the first measurement and, if possible, to refrain from using cosmetic products such as make-up, shampoo or perfume.

At 08:00, participants complete questionnaires (36-Item Short Form Health Survey (SF-36), International Physical Activity Questionnaire (IPAQ)) and undergo bioimpedance body composition analysis (BIA; InBody 770). HbA1c is measured on site only if no recent result (<3 months) is available from the patient record, using a DCA Vantage analyser (Siemens) according to the manufacturer’s instructions. Two fasting breath samples and one capillary glucose measurement are then collected. Participants subsequently provide an oral microbiota swab and consume a standardised breakfast (YFood shake providing 200 kcal: 15 g carbohydrates, 12.5 g protein, 8 g fat and 3 g fibre).

**Figure 1:**
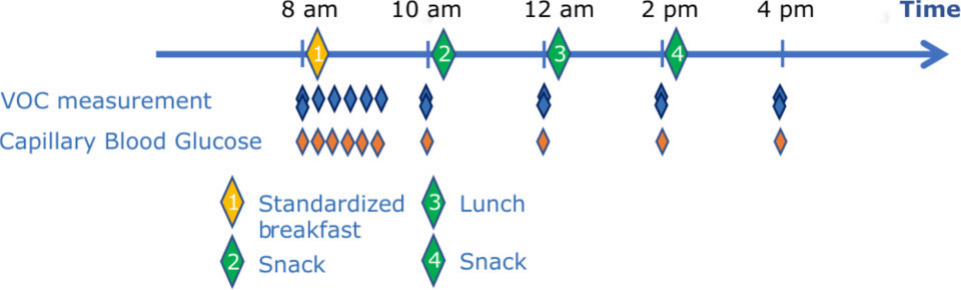
Schematic measurement schedule. Participants arrive fasting at 08:00, complete baseline assessments and consume a standardised breakfast. Breath VOC and capillary glucose samples are collected at fasted baseline and every ∼15 min during the first postprandial period. Subsequent meals (meals 2–4) are not standardised and are consumed voluntarily; breath VOC and glucose samples are collected immediately before each eating episode. VOC, volatile organic compound.

Breath VOC and glucose samples are collected every 15 min for approximately 90 min. For the remainder of the day, participants may consume self-selected meals or snacks according to their usual routine, with breath VOC and glucose sampling immediately beforehand. An overview of the study schedule is illustrated in figure 1, and the schedule is summarised in table 2.

**Table 2:** Schedule of study procedures

Procedure	Previsit	Visit day
Eligibility screening	X	
Informed consent	X	
Fasting and preparation instructions	X	
Diet and sleep diary (covering 2 days before visit)	X	Return (on or after visit)
Stool sample kit (collection within ±2 days)	X	Return (on or after visit)
Questionnaires (SF-36, IPAQ)		X
Body composition (BIA)		X
Oral microbiota swab		X
HbA1c (if not available <3 months in patient records)		X
Baseline breath VOC and capillary glucose		X
Standardised breakfast		X
VOC and glucose every 15 min (first 90 min)		X
VOC and glucose before subsequent meals/snacks		X

BIA, Bioelectrical Impedance Analysis; IPAQ, International Physical Activity Questionnaire; SF-3, 36-Item Short Form Health Survey; VOC, volatile organic compound.

### VOC and biospecimen collection

Breath samples are collected with a BreathSpec^®^ gas chromatography–ion mobility spectrometry system (GC-IMS). Capillary blood glucose is measured via fingerprick sampling using a Contour^®^ XT meter. Oral and stool samples undergo microbiota sequencing using established workflows. Raw instrument files and metadata are stored securely.

### Data sources and documentation

Study data include participant questionnaires, capillary glucose, breath VOC profiles, and oral and stool microbiota samples. All data collection instruments and source documents are summarised in table 3.

**Table 3:** Data collection and source documents

Data item	Method/device	Source
Demographics, history, medication	Self-report	eCRF
SF-36, IPAQ	Standard questionnaires	eCRF
Body composition	Bioimpedance (InBody)	Device printout
HbA1c (if needed)	DCA Vantage®	Device record
Oral microbiota	OMNIGene^TM^ Oral Kit 16S-Seq.	Sequencing report
Stool microbiota	OMNIGene^TM^ Gut Kit 16S-Seq.	Sequencing report
Capillary glucose	Contour® XT metre	Device output
Breath VOCs	BreathSpec® GC-IMS	Instrument files
Diet/sleep diaries	Participant record	Diary/eCRF

eCRF, electronic Case Report Form; GC-IMS, gas chromatography–ion mobility spectrometry system; HbA1c, Glycated haemoglobin; IPAQ, International Physical Activity Questionnaire; SF-36, 36-Item Short Form Health Survey; VOCs, volatile organic compounds.

### Data management plan

Study data, including VOC instrument files, questionnaire data, capillary glucose values and microbiota sequencing outputs, are stored in secure institutional systems with role-based access. Source documents and metadata are maintained in REDCap with audit trails.

### Outcomes

The primary outcome is the temporal profile of VOC intensity across the 08:00–16:00 period. Secondary outcomes include differences across metabolic groups, associations between VOCs and capillary glucose ranges, and relationships between VOCs, body composition and microbiota profiles. The outcomes are summarised in table 4.

**Table 4:** Outcomes

Outcome type	Description
Primary	Temporal variation in breath VOC intensities (08:00–16:00)
Secondary	Between-group comparisons (no diabetes, T1D, T2D)
	Associations between VOCs and capillary glucose
	Links between VOCs, body composition and microbiota

T1D, type 1 diabetes; T2D, type 2 diabetes; VOCs, volatile organic compounds.

### Sample size

A total of up to 60 participants will be enrolled, initially targeting approximately 10 per group, with variation in sex and BMI where possible. Recruitment aims for an approximate balance by sex and by BMI category (<25 vs ≥25 kg/m²). After around 30 participants, an interim exploratory assessment will be performed to evaluate whether discernible temporal patterns are present in the VOC data. If no circadian intensity changes are observed in at least three VOCs, the study will be stopped early; otherwise, recruitment will continue to the planned maximum of 60 participants. No formal hypothesis testing or statistical stopping rules are applied, as this is an exploratory study.

### Data processing and analysis

Data are processed using VOCal (GAS Dortmund) with retention index libraries (eg, NIST2020) and drift-time alignment. Analysis uses Python (gc-ims-tools) and R. Temporal VOC intensity profiles will be characterised descriptively across the 08:00-16:00 window, and within-subject changes over time will be assessed using non-parametric repeated-measures approaches appropriate for the data structure. Gallery plots will support visual inspection of spectral patterns. Between-group comparisons will include both overall group differences and exploratory pairwise comparisons (no diabetes vs T1D, no diabetes vs T2D, and T1D vs T2D), using appropriate non-parametric tests (eg, Wilcoxon rank-sum or Kruskal-Wallis). Given the exploratory design and limited consistency in prior findings, no single comparison is prioritised a priori. Associations between VOCs and capillary glucose, body composition and microbiota composition will be assessed using correlation analyses, including repeated-measures correlation for intraindividual relationships. For between-group VOC discrimination, supervised multivariate methods including Partial Least Squares Discriminant Analysis (PLS-DA) and Support Vector Machine (SVM) may be applied as exploratory tools, building on approaches previously used with GC-IMS breath data in this study population.^8^ Principal component analysis supports dimensionality reduction and visual exploration of VOC patterns. No imputation is planned.

### Patient and public involvement

This study was not developed through a dedicated patient advisory panel, but its research question and design were informed by continuous interactions with people living with diabetes in the clinical and research environment of the Department of Diabetes, Endocrinology, Nutritional Medicine and Metabolism and the DCB foundation. Both institutions maintain close connections with the diabetes community and regularly organise patient engagement events where individuals with lived experience provide feedback on research relevance and feasibility. Beyond the clinical setting, the research team contributes to broader public engagement through the University of Bern’s science fairs, Clinical Research Day and the national Future Day for school children. Findings from this project will be shared at future DCB patient engagement events and public science days to foster dialogue and mutual learning between researchers, patients, healthcare professionals and the wider community.

### Ethics and dissemination

The study complies with the Declaration of Helsinki and the Swiss Human Research Act/Ordinance. Ethics approval was granted by the Ethics Committee of the Canton of Bern (BASEC 2023-01143) and is registered on ClinicalTrials.gov under NCT05984979. Written informed consent is obtained from all participants before any study procedures. As this is a non-invasive observational study involving breath sampling and questionnaires, no major risks are anticipated; any adverse events will be documented.

Results will be disseminated through peer-reviewed publications, conference presentations and lay summaries. Deidentified data and analysis code will be deposited in Zenodo/Dryad and GitHub after study completion in accordance with institutional and repository policies.

## Discussion

This study examines whether daytime changes in exhaled VOCs can be observed under controlled, real-world-compatible conditions and whether such changes differ across metabolic groups. Breath signatures in diabetes have shown promise, but reproducibility challenges remain. Time-of-day effects are plausible contributors and have not been systematically explored in diabetes research. A standardised breakfast and structured sampling allow evaluation of short-term metabolic responses, while participant-selected meals later in the day preserve ecological validity.

This work is exploratory and not powered for formal hypothesis testing. Instead, the intent is to generate data on temporal variation, effect size estimates and protocol considerations for future studies. Insights regarding sampling windows, fasting requirements and links to glucose or microbiota profiles may help refine breath-based metabolic monitoring approaches. If meaningful daytime variation emerges, time-locked sampling may be warranted; if not, breath analysis may offer greater flexibility than currently assumed.

## Supplementary material

10.1136/bmjopen-2025-113892 online supplemental file 1
