# Supplementary material for: Daytime circadian patterns of exhaled volatile organic compounds in adults without and with type 1 and type 2 diabetes: protocol for an exploratory observational study
Source: BMJ Open. 2026 Apr 8;16(4):e113892. doi: 10.1136/bmjopen-2025-113892 (PMC13064124; doi:10.1136/bmjopen-2025-113892)
Supplement: online supplemental file 1 [file bmjopen-16-4-s001.docx]

STROBE Statement—checklist of items that should be included in reports of observational studies

|  | Item No. | Recommendation | Page  No. | Relevant text from manuscript |
| --- | --- | --- | --- | --- |
| **Title and abstract** | 1 | (*a*) Indicate the study’s design with a commonly used term in the title or the abstract | 1 | Title: “Daytime Circadian Patterns of Exhaled Volatile Organic Compounds in Adults Without and With Type 1 and Type 2 Diabetes: Protocol for an Exploratory Observational Study.* |
|  |  | (*b*) Provide in the abstract an informative and balanced summary of what was done and what was found | 1 | Abstract: “Introduction: Circadian regulation modulates metabolic and hormonal processes throughout the day, yet it remains unclear whether these diurnal fluctuations are reflected in exhaled volatile organic compound (VOC) profiles and whether such temporal patterns differ between individuals with and without diabetes. Previous breath analysis studies in diabetes have shown heterogeneous results, which may reflect differences in analytical approaches and the lack of standardized sampling times.  Methods and analysis: This prospective, single-centre observational study examines daytime VOC dynamics from 08:00 to 16:00 amongst adults without diabetes, and individuals with type 1 diabetes (T1D) or type 2 diabetes (T2D). Sixty participants will complete one in-person visit with repeated breath measurements using a BreathSpec GC–IMS device, capillary glucose testing, body composition assessment, questionnaires, and oral and stool microbiota sampling. A standardised breakfast is provided; subsequent meals follow structured timing but are not standardised. The primary outcome is temporal variation in VOC intensities. Secondary outcomes include between-group differences and associations with glucose levels, body composition, and microbiota composition. Analyses will use established GC–IMS tools and exploratory multivariate approaches.  Ethics and dissemination: Ethics approval was granted by the Ethics Committee of the Canton of Bern (BASEC 2023-01143). Results will be shared via peer-reviewed publications, conferences, and lay summaries.  Trial registration number: ClinicalTrials.gov Identifier: NCT05984979.” |
| Introduction | | | |  |
| Background/rationale | 2 | Explain the scientific background and rationale for the investigation being reported | 2 | Introduction: “Breath volatile organic compounds (VOCs) arise from endogenous metabolism, microbial activity, and environmental exposures, and have been evaluated as noninvasive markers in metabolic and inflammatory contexts… Circadian physiology may also influence breath VOC patterns… Yet, in diabetes research, sampling times are rarely standardized.” |
| Objectives | 3 | State specific objectives, including any prespecified hypotheses | 2 | Introduction, final paragraph: “The aim is to characterize temporal patterns in exhaled VOCs in adults with type 1 and type 2 diabetes and those without diabetes, and to explore associations with glucose, body composition, and oral and stool microbiota.” |
| Methods | | | |  |
| Study design | 4 | Present key elements of study design early in the paper | 3 | Methods and analysis – Study design and setting: “This prospective, single-center observational study is conducted in the clinical study facilities at the Diabetes Center Berne (DCB), Switzerland. Each participant completes one eight-hour study visit.” |
| Setting | 5 | Describe the setting, locations, and relevant dates, including periods of recruitment, exposure, follow-up, and data collection | 3 | Methods and analysis – Study design and setting: “This prospective, single-center observational study is conducted in the clinical study facilities at the Diabetes Center Berne (DCB), Switzerland. Each participant completes one eight-hour study visit.”  and  Study status and timeline: “Recruitment began in 2023, with data collection completed for three participants. The study has been paused since June 2024 due to device unavailability and resumed in December 2025. No interim analyses have been conducted to date. Enrolment is expected to conclude by October 2026, with primary data analysis completed within approximately 6 months thereafter.” |
| Participants | 6 | (*a*) *Cohort study*—Give the eligibility criteria, and the sources and methods of selection of participants. Describe methods of follow-up  *Case-control study*—Give the eligibility criteria, and the sources and methods of case ascertainment and control selection. Give the rationale for the choice of cases and controls  *Cross-sectional study*—Give the eligibility criteria, and the sources and methods of selection of participants | 3 | Participants and eligibility: “Three adult groups are enrolled: individuals without diabetes, those with type 1 diabetes (T1D), and those with type 2 diabetes (T2D)... Key exclusions include pregnancy or breastfeeding; smoking within six months; chronic pulmonary or intestinal disease…” |
|  |  | (*b*) *Cohort study*—For matched studies, give matching criteria and number of exposed and unexposed  *Case-control study*—For matched studies, give matching criteria and the number of controls per case |  |  |
| Variables | 7 | Clearly define all outcomes, exposures, predictors, potential confounders, and effect modifiers. Give diagnostic criteria, if applicable | 6 | Outcomes section & Table 4: “Primary outcome: temporal variation in breath VOC intensities (08:00–16:00). Secondary outcomes include between-group comparisons and associations with glucose, body composition, and microbiota.” |
| Data sources/ measurement | 8* | For each variable of interest, give sources of data and details of methods of assessment (measurement). Describe comparability of assessment methods if there is more than one group | *5,6* | Data sources and documentation & Table 3: “Breath samples are collected with a BreathSpec gas chromatography–ion mobility spectrometry system. Capillary glucose is measured using a Contour XT meter. Oral and stool samples undergo microbiota sequencing using established workflows.” |
| Bias | 9 | Describe any efforts to address potential sources of bias | 1 | Strengths and limitations of this study: “Controlled yet pragmatic single-day design enables assessment of daytime variation in exhaled VOCs under near-real-world conditions.” |
| Study size | 10 | Explain how the study size was arrived at | 7 | Sample size: “A total of up to 60 participants will be enrolled... appropriate for exploratory analysis of VOC trajectories and model development in GC–IMS data.” |

Continued on next page

| Quantitative variables | 11 | Explain how quantitative variables were handled in the analyses. If applicable, describe which groupings were chosen and why | 7 | Data processing and analysis: “VOC intensity curves, gallery plots, and nonparametric tests explore temporal behavior and group differences. Correlation and repeated-measures approaches assess relationships with glucose and microbiota.” |
| --- | --- | --- | --- | --- |
| Statistical methods | 12 | (*a*) Describe all statistical methods, including those used to control for confounding | 7 | Data processing and analysis: “Temporal VOC intensity profiles will be characterised descriptively across the 08:00-16:00 window, and within-subject changes over time will be assessed using non-parametric repeated-measures approaches appropriate for the data structure. Gallery plots will support visual inspection of spectral patterns. Between-group comparisons will include both overall group differences and exploratory pairwise comparisons (no diabetes vs. T1D, no diabetes vs. T2D, and T1D vs. T2D), using appropriate non-parametric tests (e.g., Wilcoxon rank-sum or Kruskal--Wallis). Given the exploratory design and limited consistency in prior findings, no single comparison is prioritised a priori. Associations between VOCs and capillary glucose, body composition, and microbiota composition will be assessed using correlation analyses, including repeated-measures correlation for intra-individual relationships. For between-group VOC discrimination, supervised multivariate methods including PLS-DA and SVM may be applied as exploratory tools, building on approaches previously used with GC-IMS breath data in this study population. Principal component analysis supports dimensionality reduction and visual exploration of VOC patterns. No imputation is planned.” |
|  |  | (*b*) Describe any methods used to examine subgroups and interactions | 7 | Data processing and analysis: “Temporal VOC intensity profiles will be characterised descriptively across the 08:00-16:00 window, and within-subject changes over time will be assessed using non-parametric repeated-measures approaches appropriate for the data structure. Gallery plots will support visual inspection of spectral patterns. Between-group comparisons will include both overall group differences and exploratory pairwise comparisons (no diabetes vs. T1D, no diabetes vs. T2D, and T1D vs. T2D), using appropriate non-parametric tests (e.g., Wilcoxon rank-sum or Kruskal--Wallis). Given the exploratory design and limited consistency in prior findings, no single comparison is prioritised a priori. Associations between VOCs and capillary glucose, body composition, and microbiota composition will be assessed using correlation analyses, including repeated-measures correlation for intra-individual relationships. For between-group VOC discrimination, supervised multivariate methods including PLS-DA and SVM may be applied as exploratory tools, building on approaches previously used with GC-IMS breath data in this study population. Principal component analysis supports dimensionality reduction and visual exploration of VOC patterns. No imputation is planned. |
|  |  | (*c*) Explain how missing data were addressed |  | Not applicable—protocol stage; data collection ongoing. |
|  |  | (*d*) *Cohort study*—If applicable, explain how loss to follow-up was addressed  *Case-control study*—If applicable, explain how matching of cases and controls was addressed  *Cross-sectional study*—If applicable, describe analytical methods taking account of sampling strategy |  | Not applicable—protocol stage; data collection ongoing. |
|  |  | (*e*) Describe any sensitivity analyses |  | Not applicable |
| Results | | | | |
| Participants | 13* | (a) Report numbers of individuals at each stage of study—eg numbers potentially eligible, examined for eligibility, confirmed eligible, included in the study, completing follow-up, and analysed |  | Not applicable—protocol stage; data collection ongoing. |
|  |  | (b) Give reasons for non-participation at each stage |  | Not applicable—protocol stage; data collection ongoing. |
|  |  | (c) Consider use of a flow diagram |  | Not applicable—protocol stage; data collection ongoing. |
| Descriptive data | 14* | (a) Give characteristics of study participants (eg demographic, clinical, social) and information on exposures and potential confounders |  | Not applicable—protocol stage; data collection ongoing. |
|  |  | (b) Indicate number of participants with missing data for each variable of interest |  | Not applicable—protocol stage; data collection ongoing. |
|  |  | (c) *Cohort study*—Summarise follow-up time (eg, average and total amount) |  | Not applicable—protocol stage; data collection ongoing. |
| Outcome data | 15* | *Cohort study*—Report numbers of outcome events or summary measures over time |  | Not applicable—protocol stage; data collection ongoing. |
|  |  | *Case-control study—*Report numbers in each exposure category, or summary measures of exposure |  | Not applicable—protocol stage; data collection ongoing. |
|  |  | *Cross-sectional study—*Report numbers of outcome events or summary measures |  | Not applicable—protocol stage; data collection ongoing. |
| Main results | 16 | (*a*) Give unadjusted estimates and, if applicable, confounder-adjusted estimates and their precision (eg, 95% confidence interval). Make clear which confounders were adjusted for and why they were included |  | Not applicable—protocol stage; data collection ongoing. |
|  |  | (*b*) Report category boundaries when continuous variables were categorized |  | Not applicable—protocol stage; data collection ongoing. |
|  |  | (*c*) If relevant, consider translating estimates of relative risk into absolute risk for a meaningful time period |  | Not applicable—protocol stage; data collection ongoing. |

Continued on next page

| Other analyses | 17 | Report other analyses done—eg analyses of subgroups and interactions, and sensitivity analyses |  | Not applicable—protocol stage; data collection ongoing. |
| --- | --- | --- | --- | --- |
| Discussion | | | | |
| Key results | 18 | Summarise key results with reference to study objectives | 8 | Discussion, paragraph 1: “This study examines whether daytime changes in exhaled VOCs can be observed under controlled, real-world-compatible conditions and whether such changes differ across metabolic groups.” |
| Limitations | 19 | Discuss limitations of the study, taking into account sources of potential bias or imprecision. Discuss both direction and magnitude of any potential bias | 1 | Strengths and limitations of this study: “Exploratory nature and modest sample size limit formal inference; findings will primarily inform future powered studies.” |
| Interpretation | 20 | Give a cautious overall interpretation of results considering objectives, limitations, multiplicity of analyses, results from similar studies, and other relevant evidence | 8 | Discussion: “This work is exploratory and not powered for formal hypothesis testing... If meaningful daytime variation emerges, time-locked sampling may be warranted.” |
| Generalisability | 21 | Discuss the generalisability (external validity) of the study results | 8 | Discussion: “This work is exploratory and not powered for formal hypothesis testing. Instead, the intent is to generate data on temporal variation, effect size estimates, and protocol considerations for future studies.” |
| Other information | |  | | |
| Funding | 22 | Give the source of funding and the role of the funders for the present study and, if applicable, for the original study on which the present article is based | 9 | Funding Statement: “Supported by DCB Research AG, Burgergemeinde Bern, and internal funding from the University of Bern. The funders have no role in study design, data collection, analysis, or decision to publish.” |

*Give information separately for cases and controls in case-control studies and, if applicable, for exposed and unexposed groups in cohort and cross-sectional studies.

**Note:** An Explanation and Elaboration article discusses each checklist item and gives methodological background and published examples of transparent reporting. The STROBE checklist is best used in conjunction with this article (freely available on the Web sites of PLoS Medicine at http://www.plosmedicine.org/, Annals of Internal Medicine at http://www.annals.org/, and Epidemiology at http://www.epidem.com/). Information on the STROBE Initiative is available at www.strobe-statement.org.
