# Supplementary material for: Daytime circadian patterns of exhaled volatile organic compounds in adults without and with type 1 and type 2 diabetes: protocol for an exploratory observational study
Source: BMJ Open. 2026 Apr 8;16(4):e113892. doi: 10.1136/bmjopen-2025-113892 (PMC13064124; doi:10.1136/bmjopen-2025-113892)
Supplement: Supplementary file 2 [file bmjopen-2025-113892.reviewer_comments.pdf]

PEER REVIEW HISTORY

BMJ Open publishes all reviews undertaken for accepted manuscripts. Reviewers are asked to complete a checklist review form and are provided with free text boxes to elaborate on their assessment. These free text comments are reproduced below.

ARTICLE DETAILS

Title (Provisional)

Daytime Circadian Patterns of Exhaled Volatile Organic Compounds in Adults Without and With Type 1 and Type 2 Diabetes: Protocol for an Exploratory Observational Study

Authors

Nicolier, Cléo; Rothenbühler, Marc; Balmer, Maria Luisa; Witthauer, Lilian

---

VERSION 1 - REVIEW

**Reviewer**	**1**
**Name**	**Koyama, Alain**
**Affiliation**	**Centers for Disease Control and Prevention**
**Date**	**19-Mar-2026**
**COI**	**None**

The authors describe a study protocol for an observational study investigating daytime variations of breath VOC profiles among adults with and without diabetes. I have the following comments/suggestions:

From a broad clinical and/or public health perspective, what do the authors hope that analyzing breath VOCs can advance in terms of aspects such as the etiology, prevention and/or treatment of type 1 and type 2 diabetes? Are there prior studies suggesting that breath VOCs could be a superior marker for diagnosis, prediction of disease progression, etc?

For the between group comparisons, is the aim to have pairwise comparisons for each possible comparison between the three groups? Although the study is exploratory, does prior research inform an emphasis on any particular comparison(s)?

Intercurrent infections are listed as a potential confounder – does this refer to a history of intercurrent infections, since one of the exclusion criteria is an acute infection?

In the “Data processing and analysis” section, little detail is provided as far as which tests will be conducted on which variables – are preliminary findings meant to drive the exact analyses done, given the exploratory nature of the study?

VERSION 1 - AUTHOR RESPONSE

Reviewer #1

Comment 1 — Clinical/public health relevance

From a broad clinical and/or public health perspective, what do the authors hope that analyzing breath VOCs can advance…? Are there prior studies suggesting that breath VOCs could be a superior marker…?

We thank the reviewer for this important point. We agree that the broader clinical and public health relevance should be more clearly articulated. We have expanded the Introduction n Lines 58-63 to better contextualise the potential of breath VOC analysis as a non-invasive metabolic monitoring tool, including its potential role in disease stratification, real-time metabolic assessment, and complementing existing biomarkers. We also clarify that, while promising, current evidence remains heterogeneous and does not yet establish superiority over existing diagnostic approaches:
«Despite these limitations, breath VOC analysis holds potential as a non-invasive adjunct to existing biomarkers, offering real-time metabolic information relevant to glycaemic monitoring, early detection of dysglycaemic states, disease stratification, and potentially early identification of metabolic dysregulation in populations at risk for or with undiagnosed type 2 diabetes (11,12), though superiority over established markers has not yet been demonstrated.»

Comment 2 — Between-group comparisons

Is the aim to have pairwise comparisons…? Does prior research inform emphasis?

We thank the reviewer for this helpful clarification. The study is designed to explore both global and pairwise differences across the three groups (no diabetes, T1D, T2D). Given the exploratory nature and limited prior evidence, no single comparison is prioritised a priori. We have clarified this in the Methods section lines 211-216:
« Between-group comparisons will include both overall group differences and exploratory pairwise comparisons (no diabetes vs. T1D, no diabetes vs. T2D, and T1D vs. T2D), using appropriate non-parametric tests (e.g., Wilcoxon rank-sum or Kruskal--Wallis). Given the exploratory design and limited consistency in prior findings, no single comparison is prioritised a priori.

Comment 3 — Intercurrent infections

Does this refer to history vs acute infection?

We thank the reviewer for highlighting this ambiguity. We clarify that acute infections are an exclusion criterion, whereas recent or mild intercurrent conditions not meeting exclusion criteria may still act as potential confounders and will be documented if known. Adjustments were made on lines 133-134:
«Potential confounders (e.g., recent diet, sleep, physical activity, recent or mild intercurrent infections not meeting exclusion criteria, medication changes, and environmental exposures) are documented via diaries and questionnaires for descriptive adjustment in analyses»

Comment 4 — Statistical analysis detail

Little detail is provided… are analyses driven by preliminary findings?

We thank the reviewer for this important point. We have expanded the Data processing and analysis section to clarify the analytical strategy. While predefined analytical approaches are outlined, the exploratory nature of the study allows for data-driven refinement of analyses, particularly in multivariate modelling. Lines 208-222 were adjusted accordingly:

«Temporal VOC intensity profiles will be characterised descriptively across the 08:00-16:00 window, and within-subject changes over time will be assessed using non-parametric repeated-measures approaches appropriate for the data structure. Gallery plots will support visual inspection of spectral patterns. Between-group comparisons will include both overall group differences and exploratory pairwise comparisons (no diabetes vs. T1D, no diabetes vs. T2D, and T1D vs. T2D), using appropriate non-parametric tests (e.g., Wilcoxon rank-sum or Kruskal--Wallis). Given the exploratory design and limited consistency in prior findings, no single comparison is prioritised a priori. Associations between VOCs and capillary glucose, body composition, and microbiota composition will be assessed using correlation analyses, including repeated-measures correlation for intra-individual relationships. For between-group VOC discrimination, supervised multivariate methods including PLS-DA and SVM may be applied as exploratory tools, building on approaches previously used with GC-IMS breath data in this study population (8). Principal component analysis supports dimensionality reduction and visual exploration of VOC patterns. No imputation is planned. »
